# Advanced practice radiation therapists: an Australian context

**DOI:** 10.1002/jmrs.280

**Published:** 2018-05-10

**Authors:** Bronwyn Hilder, Pieter VanDam, Kathleen Doherty

**Affiliations:** ^1^ School of Medicine, University of Tasmania Hobart Tasmania Australia; ^2^ Royal Hobart Hospital Hobart Tasmania Australia; ^3^ Wicking Dementia Research & Education Centre University of Tasmania Hobart Tasmania Australia

**Keywords:** Advanced practice, boundaries/roles, discipline, professional, radiotherapy (radiation therapy)

## Abstract

The purpose of this scoping review is to examine the literature regarding the development, implementation, scope and extent of Advanced Practice Radiation Therapist (APRT) roles in Australia in peer reviewed journals, government reports, conference proceedings and reports. A search was undertaken of PubMed, Web of Science and CINAHL, the ASMIRT website and, and Google Scholar to identify relevant documents. Combinations of keywords with Boolean operators ((advanced practice) OR (advanced practitioner) OR (specialist)) AND ((radiation therapist) OR (radiation therapy)) were used. Online and physical searches were conducted between July 16 and 23 2017. Results were not date limited. The searches retrieved 352 after duplicates were removed with 46 remaining after filtering for eligibility criteria. Items consisted of journal articles, conference abstracts, presentation slides, online presentations, State government and ASMIRT reports. A number of potential and existing APRT roles were found in the identified articles, including image review, stereotactic, treatment review, breast localisation, palliative radiotherapy, brachytherapy, radiation engineering and urology. Despite reports indicating that radiation therapists in Australia have been concerned with professional directions since 2001, there is little evidence of formal progress towards defined APRT roles. Several centres have implemented roles in a number of practice areas. The success of APRT roles lies in the ability to demonstrate that implementation goals have been achieved and that patient care has improved. The literature suggests that this is occurring, however, the presented evidence is not compelling.

## Introduction

The incidence of cancer in Australia is increasing with new diagnoses rising from 124,465 in 2013 to an estimated 134,174.^1^ Coupled with this, a growing number of people survive their initial cancer diagnosis, but “live with recurrent cancer, requiring ongoing monitoring, treatment, care and support”.^2^ This growing demand will put increased pressure on the health system, and in particular on cancer services. Radiation therapy is a safe, highly effective treatment for many types of cancer for both cure and palliation.^3^ Traditionally, planning, delivery and quality assurance of radiation therapy in the radiation oncology department has been compartmentalised, where tasks are apportioned to radiation oncologists (RO), radiation therapists (RT) and radiation oncology medical physicists (ROMP), based on their specific areas of expertise. This could lead to gaps or delays in service meaning that patients may not receive care as efficiently as possible.

One approach to improve continuity of services delivered to patients is to minimise the divide between professional groups through skills transfer. Smith et al.^4^ note that “skills transfer…has been proposed as a means of meeting growing demand” and suggests delegation of tasks traditionally completed by one professional group to another. In the context of the medical radiation science professions, Smith further argues that this delegation requires the development of advanced practice roles. Advanced practice (AP) is “assumed to indicate working beyond one's traditional scope of practice underpinned by expert evidence based knowledge”.^5^

Advanced Practice Radiation Therapist (APRT) positions have been in place for more than a decade in the United Kingdom^6^ and the province of Ontario^7^ in Canada. These roles were developed in response to drivers such as increased demands for service and emerging technologies and are underpinned by increased autonomy in RT practice.^6^ The key goal for the implementation of APRT roles is to improve service delivery for patients receiving radiation therapy treatment, including improved access, timeliness and treatment quality.^8^

There is evidence that APRT roles have been developed and implemented in Australia, however, little has been published regarding these roles. There is now a pathway for individual recognition by the professional body, the Australian Society of Medical Imaging and Radiation Therapy (ASMIRT, formerly known as the Australian Institute of Radiography, AIR) and there are currently four professionally recognised APRT in Australia: two in breast, one in urogenital and one in palliation.

The purpose of this scoping review is to examine the development, implementation, scope and extent of APRT roles in Australia in peer reviewed journals, government and professional body reports and conference proceedings. An understanding of the current state of these roles will assist in answering the question of whether APRT roles can play a part in addressing the increasing demands on radiotherapy services and improve patient outcomes.

## Method

A search was undertaken of the databases PubMed, Web of Science and CINAHL to identify literature relating to APRT roles in Australia. The “view related/similar article/cited by” features of each of the databases were also used. A search of the ASMIRT website, ASMIRT conference handbooks and Google Scholar was also conducted to identify other relevant documents or reports published from other sources. Combinations of keywords with Boolean operators (*advanced practice*) OR (*advanced practitioner*) OR (*specialist*) AND (*radiation therapist*) OR (*radiation therapy*) were used. Searches and physical searches were conducted between July 16 and 23, 2017. Results were not date limited and included the date of the search. To increase the likelihood of identifying relevant literature, citations and references within the documents retrieved in the initial search were also reviewed. The search was restricted to literature published in English.

Eligibility criteria included information regarding the development, implementation, scope and extent of APRT roles in Australia with source as a secondary criteria to determine inclusion. Articles must have been published in peer reviewed journals providing data regarding APRT roles in Australia. Reports must have been released under the auspices of state or federal governments or professional bodies representing RTs. Studies discussing role expansion for RTs were included as evidence of progress towards implementation. Abstracts of conference presentations, conference e‐posters, editorials and commentary were included where it was judged to have relevance to the purpose of the review.

Titles and abstracts of journal articles and report summaries were reviewed to determine eligibility for inclusion. If it was unclear that an article, report, editorial or commentary met eligibility criteria, it was retrieved and viewed in full prior to inclusion. Editorials and commentaries were viewed in full. Conference presentations were included where complete abstracts were published or presentations were available to view. The abstracts of e‐posters were reviewed and if eligible were retrieved. Whilst these latter sources were judged to be of less weight when compared to those higher in the evidence hierarchy such as peer reviewed journal articles, they were included to provide context in light of the scarcity of literature.

**Figure 1:**
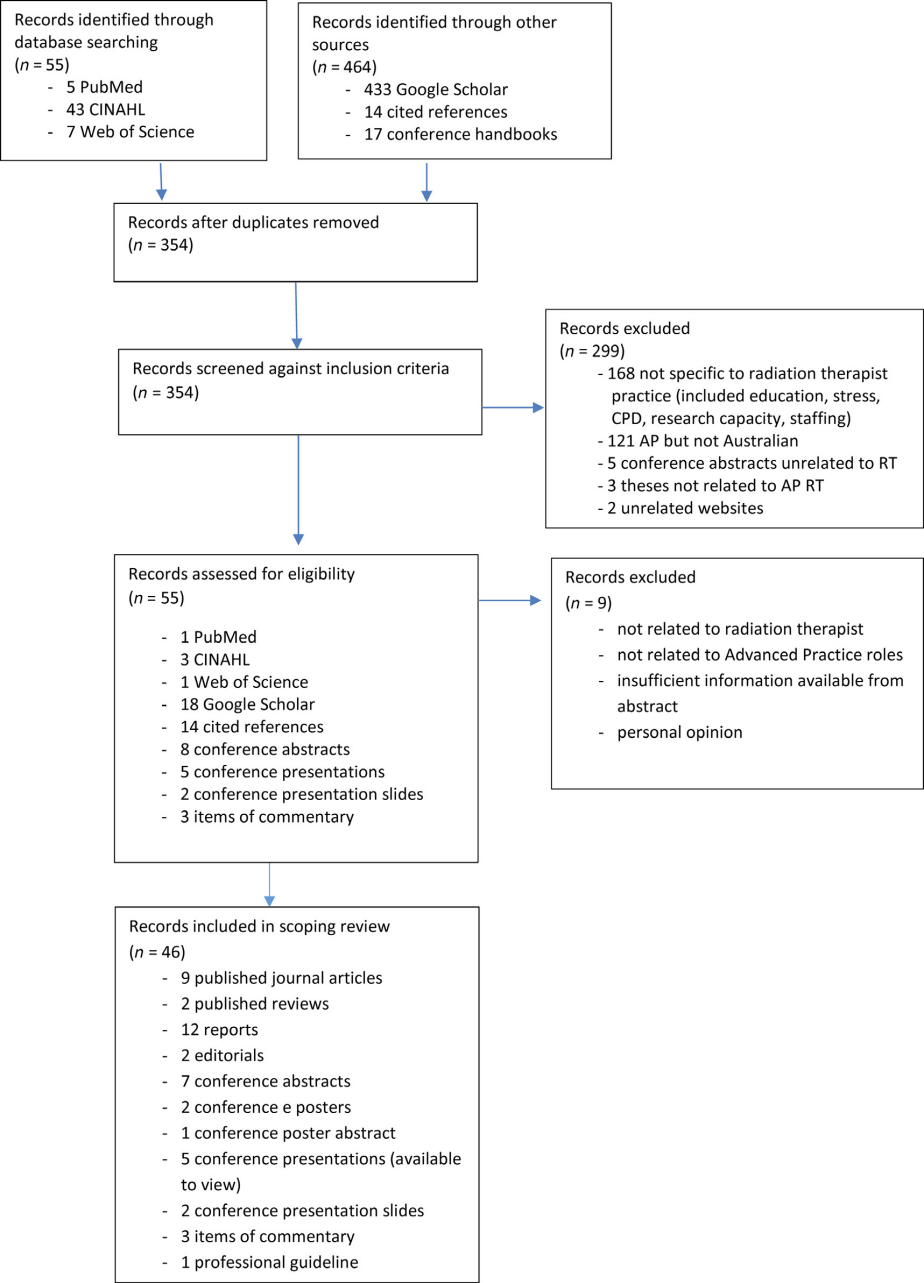
Flowchart for search strategy and selection process for a review of literature of advanced practice radiation therapist roles in Australia.

A flowchart was constructed to document the search strategy and record the items found, reasons for exclusion and subsequent number of items that met the inclusion criteria (Fig. 1). These items were documented in a summary table (Table 1). After the initial search all subsequently identified items were checked against the summary table to exclude duplicates. The table was updated throughout the search process, resulting in a summary of all relevant items for the review.

**Table 1:** Chronological list of relevant literature

Year	Author	Type	Title	Notes
2006	Australian Institute of Radiography	Report	Professional Advancement Working Party Report (39)	First report by the professional body into advanced practice models for Australia
2007	Ahern, Bull, Harris, Matthews, Willis	Editorial	Subspecialisation of radiation therapists in Australia and New Zealand (37)	Suggested specialised RT role for paediatric patients
2008	Smith, Yielder, Ajibulu, Caruana	Review article	Progress towards advanced practice roles in Australia, New Zealand and the Western Pacific (4)	Review of AP roles in diagnostic and radiation therapy – limited number and scope
2008	Rybovic, Halkett, Banati & Cox	Journal article	Radiation therapists’ perceptions of the minimum level of experience required to perform portal image analysis (11)	Research conducted in early implementation of online image review
2009	Advanced Practice Working Group	Report	Discussion paper: a model of advanced practice in diagnostic imaging and radiation therapy in Australia	Follow‐up report by the professional body advancing a model for AP in Australia
2009	ACT Health	Report	A systematic review of the literature: extended scope of practice radiation therapy (47)	Report of literature for extended scope of RT practice in the ACT
2009	ACT Health	Report	Current practice report: extended scope of practice radiation therapy (48)	Report on current practice in RT in the ACT
2009	ACT Health	Report	Radiation therapy extended scope of practice: phase 1 (49)	Report including suggested APRT roles in the ACT
2009	Acharya, Acharya, Vatsavayi & Cox	Review article	Systematic review – role expansion in radiation therapy:from an international perspective to an Australian context (9)	Review of role expansion for RTs, drawing from international practice to recommend AP RT roles in Australia
2009	Smith	Editorial	Advanced practice – profession‐led and patient‐focused (56)	Editorial calling for AP roles to be developed in both diagnostic radiography and radiation therapy
2009	Alfieri, Le Mottee, Arifuddin, Field, Milinkovic & Cox	Journal article	Radiation therapist‐led weekly patient treatment reviews (16)	Explores the feasibility of RT led treatment reviews, impact on service and requirements for implementation
2009	Burow, Cavenagh, Simpson, West, Cox & Szymura	Journal article	Avenues for role expansion in image guided radiation therapy: discussion and recommendations for kilovoltage and megavoltage imaging (12)	Explores requirements for specialist RT roles in image guided radiation therapy
2009	Dempsey & Burr	Journal article	The level of confidence and responsibility accepted by Australian radiation therapists in developing plans and implementing treatment (51)	RTs confident in completing plans of all levels but found barriers to accepting responsibility for plan implementation
2012	Rivett, Cooper & Brennan	Conference paper abstract	RT‐lead post‐radiotherapy treatment reviews in rural and remote settings (19)	Process used to develop APRT role in the post‐treatment follow‐up review of Head and Neck patients via telehealth ASMMIRT 2012, Sydney
2012	Freckleton	Report	Advanced practice in radiography and radiation therapy: report from the inter‐professional advisory team (41)	Recommends adoption of modified four tier model to progress AP
2012	Matthews	Conference paper abstract	Evaluation of Specialist practice radiation therapists at Peter Mac – an analysis of impact and future capacity (14)	Found APRT roles have had varying success across the organisation ASMMIRT 2012, Sydney
2012	Cox, Short & Szymura	Conference paper abstract	Radiation therapists take on greater image review responsibilities in 2011 (13)	Found that online image review had become an integral part of RT practice rather than extension or expansion ASSMIRT 2012, Sydney
2013	Australian Institute of Radiography Advanced Practice Advisory Panel	Report	APAP background report and suggested processes for implementation of IPAT recommendations (42)	Recommendations for implementation processes for AP
2013	Monk, Wrightson & Smith	Journal article	An exploration of the feasibility of radiation therapist participation in treatment reviews (17)	Feasibility measures for implementation of RT led treatment review were not met
2013	Harris	Commentary	Re: Monk CM, Wrightson SJ, Smith TN. An exploration of the feasibility of radiation therapist participation in treatment reviews. J Med Rad Sci 2013; 60: 100–7 (25)	Argues that agreement of need and evidence of capability and constant communication within the multidisciplinary team is required for APRT roles
2013	Cox	Commentary	Re: Monk CM, Wrightson SJ, Smith TN. An exploration of the feasibility of radiation therapist participation in treatment reviews. *J Med Radiat Sci* 2013; **60**: 100–7 (15)	Provides further evidence to support APRT roles in treatment review
2013	Monk	Commentary	Response to letters to the editor regarding ‘feasibility of radiation therapist–performed treatment reviews’ (21)	Response to Harris and Cox. Argues that local issues impact on feasibility of roles and that professional bodies need to work together to negotiate boundaries
2013	Acharya, Cox, Rinks, Gaur & Back	Journal article	Ability of radiation therapists to assess radiation‐induced skin toxicity (18)	Found that experienced RTs could assess breast cancer skin toxicity as part of their role.
2013	Toikka	Conference paper abstract	Breast advance practice role implementation: our experience (27)	Preliminary results indicate that the introduction of an advance practice RT for breast localisation and delineation was received positively. Gains in efficiency, continuity and flexibility were supported. ASMMIRT Hobart; 2013.
2013	Job, Owen & Whiting	Conference paper abstract	Rapid response radiation therapist: an expanding role in the palliative radiation oncology service in Australia (29)	Project to develop APRT role in palliative radiation therapy ASMMIRT Hobart; 2013.
2013	Monk, Wrightson & Smith	Conference paper abstract	Exploration of the feasibility of radiation therapist‐performed treatment reviews (20)	Feasibility measures for implementation of RT led treatment review were not met ASMMIRT Hobart; 2013.
2013	Karzon	Conference paper abstract	Radiation therapist led treatment review – the art of caring/state of the art care. (22)	Exploration of requirements for treatment review APR ASMMIRT Hobart; 2013
2013	Department of Health and Human Services Victoria	Report	Advancing radiation therapy practice: a regional focus (26)	Project design for developing AP curriculum
2014	Cox, Newton, Rinks, Atyeo, Barnes & Lamoury	e poster	Are radiation therapists effective as treatment reviewers? The TORToiSe project (23)	Found RT review was a useful adjunct to RO review with benefits for both patients and the RT reviewers CSM 2014 Melbourne
2014	Newton, Cox, Davies, Rinks, Atyeo (24)	e poster	RT led treatment reviews: Where to from here? (24)	Found RTs are capable of conducting treatment reviews with the same standard of care as ROs CSM2014 Melbourne
2014	Matthews & Cunningham	Conference presentation	Evidence‐based curriculum design to support the training of advanced practitioners in radiation therapy (35)	Describes project for development of national education curriculum framework for APRT CSM 2014 Melbourne
2014	Brown	Conference presentation	Advanced practice, my journey (34)	Describes development, implementation and conduct of genitourinary APRRT role CSM 2014 Melbourne
2014	Job, Owen & Holt	Conference presentation	Assessing the ability of a radiation therapist to delineate simple palliative radiation therapy fields (30)	Found concordance of field placement between RO and palliative APRT CMS 2014 Melbourne
2014	Australian Institute of Radiography Advanced Practice Advisory Panel	Report	Pathway to advanced practice. (43)	Outlines the professional bodies view of the pathway to advanced practice
2014	Australian Institute of Radiography. Advanced Practice Advisory Panel	Report	Pathway to advanced practice: summary document and guidelines for application for accreditation (44)	Outlines process for accreditation as an AP for accreditation by the professional body
2014	Matthews, Wright & Osborne	Journal article	Blending work‐integrated learning with distance education in an Australian radiation therapy advanced practice curriculum. (5)	Describes rationale for curriculum development of short courses to support AP roles
2014	Department of Health and Human Services Tasmania	Report	Governance framework for implementation of expanded scope of practice for allied health professions in the Tasmanian health system (50)	Outlines background for and framework for implementation across allied health professions, including RT
2015	Smith, Maresse, Harris, Woznitza & Sale	Journal article	Conceptualisation of the characteristics of advanced practitioners in the medical radiation professions (46)	Discusses the concepts underpinning the seven characteristics of the AP model of the professional body
2015	Foote, Bailey, Smith, Siva, Hegi‐Johnson, Seeley, Barry, Booth, Ball & Thwaites	Guideline	Guidelines for safe practice of stereotactic body (ablative) radiation therapy (38)	Multidisciplinary practice guideline advocating APRT roles for stereotactice body radiation therapy.
2015	Job, Holt & Whiting	Conference presentation	Rapid referrals: reducing the wait times for palliative patients (31)	Scope of practice for palliative APRT NZIMRT‐AIR Scientific Meeting, 2015 Wellington
2016	Matthews	Conference slides	Australian radiation therapy advanced practice: a focus group study (36)	Outline of project to understand factors influencing implementation and practice of APRT roles in Australia LTWRAP 2016
2016	Matthews	Conference slides	Advanced practice at Peter MacCallum cancer centre: an evolving concept (28)	Overview of implementation of AP RT roles at PMCC and plan for review LTWRAP Conference 2016
2016	Sale, Halkett & Cox	Journal article	National survey on the practice of radiation therapist in Australia (10)	Survey of existing scope of practice for Australian RTs
2016	Job, & Holt	Conference presentation	Evaluation of AP RT in palliative radiation therapy (33)	Time from referral to treatment reduced when referred to APRT pathway when compared to standard referral. APRT field delineation comparable to that observed with interobserver delineation between radiation oncologists. ASMMIRT, Brisbane 2016
2016	Job & Holt	Poster Abstract	Evaluation of advanced practice radiation therapist role in palliative radiation therapy(32)	Time from referral to treatment reduced with introduction of APRT role Palliative care in oncology symposium 2016 San Francisco
2017	Australian Society of Medical Imaging and Radiation Therapy Advanced Practice Advisory Panel	Report	Pathway to advanced practice Summary document and guidelines for application for credentialing Advanced practice for the Australian medical radiation professions (45)	Updated processes reflecting closure of grandfathering pathway.

## Results

Initial searches of databases retrieved 55 items. Searching other sources including Google Scholar, conference handbooks and cited references yielded 269 additional items. After duplicates were removed, 287 items remained. Titles and abstracts of journal articles were viewed before retrieving 13 in full. Of these, two were review articles, one a conceptual paper, eight discussed elements of APRT roles and two were not relevant. Reports regarding implementation of AP and expanded practice roles were retrieved in full. Conference abstracts were retrieved from printed and online conference handbooks. In two cases, presentation slides were available for retrieval. In three cases, presentations were retrieved for online viewing. A total of 46 items were included in the review.

Acharya et al.^9^ published a systematic review on the state of role expansion internationally to understand the opportunities for such roles in Australia. This review identified six potential roles and noted that none were present in Australia at the time of publication. A survey of national practice^10^ notes that some advanced practice roles did exist but that they were locally driven and without formal structure. Another review reported that little development had been made towards advanced practice roles in Australia but elements of extended practice were being undertaken, including CT planning, verification, patient weekly reviews, and planning and delivery of brachytherapy treatment.^4^

A number of existing and proposed APRT roles were identified. These included roles such as image review,^11^, ^12^, ^13^, ^14^, ^15^ treatment review,^15^, ^16^, ^17^, ^18^, ^19^, ^20^, ^21^, ^22^, ^23^, ^24^, ^25^, ^26^ breast localisation,^14^, ^27^, ^28^ palliative^29^, ^30^, ^31^, ^32^, ^33^ and urology.^34^ Two conference abstracts note APRT roles at the Peter MacCallum Cancer Centre (PMCC), including imaging, breast, brachytherapy and radiation engineering.^14^, ^28^ All of these roles are closely allied with an article^5^ on curriculum design and a conference presentation^35^ to support AP roles. A conference presentation in 2016 indicates that research into the barriers to implementation of AP roles is currently being undertaken.^36^ A multidisciplinary group editorial^37^ proposed an APRT role in paediatric radiotherapy treatment, advocating for specialist RTs in the care of children undergoing radiation therapy. A practice guideline authored by a multidisciplinary group advocating for an APRT role in stereotactic ablative body radiation therapy to “be responsible for management of RT responsibilities within the SABR program”^38^ also met the inclusion criteria.

ASMIRT produced a number of reports relating to AP for radiographers and RTs.^39^, ^40^, ^41^, ^42^ These culminated in Advanced Practice Pathway documents in 2014^43^, ^44^ with a revision in 2017.^45^ The paper by Smith et al.^46^ underpins this model. Australian Capital Territory (ACT) Health developed 3 documents relating to extended practice for RTs, including a systematic review of the literature, a report into current practice and a phase 1 document identifying potential areas of role extension.^47^, ^48^, ^49^ No reports relating to subsequent phases were identified. In 2013, the Department of Health and Human Services, Victoria, produced a project report on APRT.^26^ In 2014, the Department of Health and Human Services (Tasmania) prepared a governance framework for implementation of expanded scope of practice for allied health professions in the Tasmanian Health System including the radiation therapy profession.^50^

## Discussion

A motion passed at the Annual General Meeting of the AIR in 2001 resulted in the formation of a steering committee to investigate *“*what we are going to be doing in 10 years time…the model they would expect and that the direction for the implementation of the model is put into place by the year 2012”.^39^ The timing coincided with the roll‐out of the four tier practitioner model in the UK. The Professional Advancement Working Party (PAWP) was charged with evaluating role extension and role expansion, identifying the feasibility of role expansion and the education required; essentially the precursors to the development of an advanced practice model. The 2006 PAWP report delineates the difference between role extension and role expansion, noting that the former is “the acquisition of additional knowledge and skills as a direct result of the increasing demands made upon the professions”^39^ whilst the latter refers to “formally and explicitly recognising enlargement of existing scope of practice into new tiers of practice accompanied by additional education, theory and practice”.^39^ The AP model was built upon the latter definition. A report by the Advanced Practice Working Group (APWG) in 2009^39^ elaborated on a model and outlined the scope for several diagnostic imaging and radiation therapy AP roles. This report also gives a clear recommendation for engagement with stakeholders, including other professional bodies, state and federal government, tertiary institutions, medical colleges and private providers, as necessary to moving forward with AP roles.

Despite interest shown in an AP model, a review published in 2009 identified a lack of data in Australia identifying or evaluating APRT roles^9^ with a further study^16^ noting little progress had been made towards formal APRT roles. Further to this, in 2009, Dempsey and Burr noted that radiation therapists were “reluctant to progress the issue of responsibility for higher level plans and treatment…..without the appointment at a senior or advanced practitioner level”.^51^ Current literature suggests that development, implementation and the establishment of APRT roles has occurred since that time. The success of any APRT role lies in the ability to demonstrate that the APRT has the requisite knowledge and skill to perform the duties of the role and that the desired goals which led to the creation of the role have been met. There is some limited evidence of the effectiveness of the roles in addressing both of these measures in the Australian literature, for example concordance measures of field placement between APRTs and ROs^30^ and decreased patient wait times.^31^

Drivers such as the growing demand for services, the expansion of radiotherapy services in regional areas and the rapidly changing technology in the professional field have provided impetus for the development of APRT roles. The APWG report notes that the focus of these roles should be primarily directed towards the needs of the patient: improving service delivery and patient care, by addressing service gaps and delays, and reducing wait times.^39^ Acharya argues that APRT role development can be seen as a means of “embracing innovative ways of service provision to maximise patient benefits and promoting flexible career pathways to retain highly skilled health practitioners”.^9^ There is, however, no published literature to support the latter in Australia.

In a survey designed to define current RT practice in 2008, Sale et al.^10^ found that “some advanced roles were currently practiced in Australia by some RTs; however, there was no evidence of structure to support these roles in the current system and they were based on local need”. Two survey respondents identified that they fulfilled advanced practice roles; one in a review clinic position and one in a physics/engineering position. Matthews wrote that “RT advanced practitioner implementation to date has been “ad‐hoc” and determined by the clinical need for such a role in individual clinical centres, hence role definition, training and scope of practice has been broadly interpreted”.^5^ However, APRT roles are intended to address identified and agreed gaps in service delivery which will differ between departments. This means that there may be “differing role descriptors and different expectations of the RTs within the roles”.^25^

The Peter MacCallum Cancer Centre (PMCC) was proactive in developing APRT roles, introducing breast localisation roles in 2006, and subsequently roles in imaging, brachytherapy and radiation engineering. These were driven by departmental needs and underpinned by distance education academic work and mentorship. PMCC at that time consisted of six sites and not all roles were present at each site. A paper presented by Matthews in 2012 evaluating the impact of the roles on stakeholders noted that the roles had had varying success across the organisation.^14^ Six of a total of 17 breast AP graduates and 14 of 24 imaging graduates were still practising in their AP role in 2016.^28^ Despite these numbers of APRTs in PMCC, there is no peer reviewed literature on the impact on service of these roles. Matthews has highlighted difficulties faced in sustaining these roles, including high attrition due to inhibition of training ability, the lack of availability of suitable academic course material, and management restructure.^28^

The abstract by Toikka in 2013 reports a positive response to the introduction of a breast APRT in her centre, supporting “gains in efficiency, continuity and flexibility”.^27^ This study evaluated the efficiencies gained in the planning process when the APRT assumed the duties of the RO in attending simulation sessions, localising breast tissue and assessing anatomical field placement on the planning CT dataset, and included measures around total simulation and planning time, resources, availability and accuracy.

The APRT roles at PMCC and other centres were supported by short course programs, developed and delivered by Monash University. Clinical mentorship from local ROs was provided to support APRT roles in breast, treatment review and advanced imaging. Approximately 50 RTs in centres across Australia had completed one of these courses in 2014.^5^ These short courses are no longer available. In 2012 a consortium of five universities was awarded funding through the Better Access to Radiation Oncology program to develop a national curriculum for APRT.^35^ This project has resulted in the development and ongoing delivery of online professional practice modules, such as advanced anatomy, psychosocial care, imaging and patient assessment and toxicity management. There is no peer reviewed evidence of their evaluation and effectiveness nor any evidence demonstrating that these modules have been used to contribute to the establishment of new APRT roles.

One of the complexities of this issue is that, over time, tasks initially seen as role expansion have become part of normal scope of practice. This is the case for the role suggested by Rybovic et al. in 2007^11^ and Burow et al in 2009.^12^ By 2011, image review had become part of normal scope of practice for RTs^13^. Matthews notes that at the PMCC there have been 24 graduates of an Advanced Imaging program with 14 still practising. The scope of these roles is much broader than image review and includes technique development, implementation, support and analysis.^28^ No evidence on the impact of service delivery, satisfaction of patients or staff with respect to these roles has been published.

The treatment review APRT role was discussed by several authors. This role has been established in a number of Trusts in the UK for many years. Alfieri found evidence to support “very positive outcomes for patients including improved communication, decreased patient waiting times and a more consistent approach to the monitoring and management of the patient through the increased continuity of care”.^16^ This is supported in peer reviewed literature from the UK and the role was found to be beneficial for RTs, improving job satisfaction and confidence through increased involvement in patient care and increased autonomy.^52^, ^53^, ^54^ The evidence for this was gained through self‐report and survey. Rivett”s^19^ abstract describes the process used to develop an AP role in post‐treatment head and neck patient review in a regional area, utilising telehealth for patients with lower acuity. The abstract by Karzon^22^ outlined the role of the AP treatment review RT at the St George Cancer Centre. Other authors investigated RT capability with respect to breast treatment review in two centres,^18^, ^23^, ^24^ including concordance between RTs and ROs in assessing skin toxicity, and patient experiences, supporting the ability of RTs to conduct treatment review in this site. Whilst providing some evidence, the study sizes are small, making it difficult to draw definite conclusions.

In 2013, Monk et al published a feasibility study of RT led treatment review in a regional radiotherapy department.^17^ This study used two measures of feasibility; a medical intervention (MI) rate of less than 35% (based on previous studies) and levels of agreement between ROs and RTs on RT capabilities to conduct treatment reviews approaching 100%. Neither measure was met, with a higher overall MI rate of 59% and agreement not approaching 100%. Furthermore, none of the five ROs were willing to delegate the review clinics to RTs. This study prompted correspondence from several writers. The first of these pointed out differences between this and other studies and presenting additional studies which support treatment review APRT roles.^15^ The second, from a chief radiation therapist in a centre which has implemented several specialist RT and one APRT role stresses the importance of inter‐professional communication, and consultation, particularly with ROs, in developing APRT roles.^25^

Research on the palliative APRT role at the Mater Radiation Oncology Centre has provided data on capability and service improvement. A retrospective blinded comparison of field placement between the APRT and RO,^30^ was followed by a prospective study where the APRT delineated the fields on DRR blinded to the RO field placement. These studies demonstrated a high level of concordance between the APRT and the RO, with 89% of fields deemed acceptable in the second study. Management by the APRT as opposed to the standard pathway has been shown to demonstrate improved mean time for the complete planning process,^29^ and reduced wait times for palliative patients.^31^, ^55^

In 2014, the AIR established a pathway for recognition as an Advanced Practitioner. This requires the submission of a practice portfolio for independent assessment by experts. The portfolio must demonstrate that the candidate meets the seven characteristics of AP and provide evidence of their advanced capability in each: expert communication; internal and external collaboration, high degree of professionalism, advanced clinical expertise, high level of scholarship and teaching, professional judgement based on evaluation of evidence and clinical situation and clinical leadership.^45^ The underpinning principles are similar to those for APRTs in the UK and for CSRTs in Ontario. In 2015, the AIR awarded the status of Advanced Practitioner to the first APRT. Since that time, a further three RTs have been awarded this status by the professional body. Whilst recognition by the professional body represents progress in advancing the overall recognition of APRT roles, there is no registration category for Advanced Practitioners with the regulatory body, the Medical Radiation Practitioners Board of Australia.

## Conclusion

The success of APRT roles in Australia lies in the ability to demonstrate that the goals of implementation have been achieved: that the gaps in service have been addressed, that service delivery, and patient care has been improved. The literature provides limited evidence that this is occurring, however, the majority is provided through conference presentations rather than papers in peer reviewed journals. Matthews noted that implementation in Australia had been “irregular and uninformed by evidence”.^35^ This lack of breadth and depth of evidence may in part be due to the emerging nature of the roles. It is important that those centres who have established APRT roles provide information about the creation and implementation of the roles, and evidence of outcome measures which support their ongoing presence. To truly harness the potential benefits of these roles, more evidence regarding timeliness of care, improvement in patient care, improved access and reduced wait times is needed to support their realisation on a national scale.

## Conflict of Interest

The authors declare no conflict of interest.
